# Maternal Low Volume Circulation Relates to Normotensive and Preeclamptic Fetal Growth Restriction

**DOI:** 10.3389/fmed.2022.902634

**Published:** 2022-06-09

**Authors:** Wilfried Gyselaers, Christoph Lees

**Affiliations:** ^1^Department of Obstetrics, Ziekenhuis Oost Limburg, Genk, Belgium; ^2^Department of Physiology, Hasselt University, Hasselt, Belgium; ^3^Centre for Fetal Care, Queen Charlotte’s and Chelsea Hospital, Imperial College Healthcare NHS Trust, London, United Kingdom; ^4^Department of Metabolism, Digestion and Reproduction, Institute for Reproductive and Developmental Biology, Imperial College London, London, United Kingdom; ^5^Department of Development and Regeneration, KU Leuven, Leuven, Belgium; ^6^Centre for Fetal Care, Queen Charlotte’s and Chelsea Hospital, London, United Kingdom

**Keywords:** maternal hemodynamic changes in pregnancy, fetal growth, intravascular volume, cardiac output, venous hemodynamics, vascular resistance, body water volume

## Abstract

This narrative review summarizes current evidence on the association between maternal low volume circulation and poor fetal growth. Though much work has been devoted to the study of cardiac output and peripheral vascular resistance, a low intravascular volume may explain why high vascular resistance causes hypertension in women with preeclampsia (PE) that is associated with fetal growth restriction (FGR) and, at the same time, presents with normotension in FGR itself. Normotensive women with small for gestational age babies show normal gestational blood volume expansion superimposed upon a constitutionally low intravascular volume. Early onset preeclampsia (EPE; occurring before 32 weeks) is commonly associated with FGR, and poor plasma volume expandability may already be present before conception, thus preceding gestational volume expansion. Experimentally induced low plasma volume in rodents predisposes to poor fetal growth and interventions that enhance plasma volume expansion in FGR have shown beneficial effects on intrauterine fetal condition, prolongation of gestation and birth weight. This review makes the case for elevating the maternal intravascular volume with physical exercise with or without Nitric Oxide Donors in FGR and EPE, and evaluating its role as a potential target for prevention and/or management of these conditions.

## Introduction

For the past 50 years, defective placentation and inadequate adaptation of uterine spiral arteries have been considered the key role players in the so-called obstetrical syndromes: preeclampsia (PE), fetal growth restriction (FGR), preterm labor, preterm premature rupture of the membranes, late spontaneous abortion, and abruptio placentae (1). Placental malperfusion and subsequent oxidative stress are associated with the impairment of both maternal and fetal systemic physiologic functions and, eventually, maternal disease, and/or fetal distress (2). In recent years, however, the focus of research into the origins of gestational hypertensive disorders with or without FGR has shifted from the placenta to maternal cardiovascular dysfunction, present before conception or developing during the earliest stages of placentation (3, 4). The interplay between maternal hemodynamics and the placentation process explains the two hemodynamic phenotypes of PE – one with high cardiac output (CO) and low vascular resistance and another with low CO and high vascular resistance – (5, 6), as well as FGR (7), and also links maternal low CO to fetal increased umbilical and reduced cerebral Doppler impedance (8). Next to this, suboptimal plasma volume expansion is considered an intrinsic pathophysiologic feature of PE with FGR (9), and maternal normotensive low volume circulation has been associated with neonatal birthweight of <10th percentile (10). The feasibility and clinical relevance of non-invasive assessment of maternal body water volumes, in association with cardiovascular assessments in both latent and symptomatic stages of PE and/or FGR, have been reported (11, 12), offering an opportunity for volume expansion strategies as potential management options in the prevention and treatment of poor fetal growth.

This narrative literature review aimed to summarize reported evidence on the association between poor fetal growth and low maternal circulating volume as a constitutional predisposing condition in normotensive FGR or as a consequence of suboptimal volume expansion in PE with FGR. The methodology was performed according to Sandra’s principles (13) using the following keywords (alone or in combination): FGR, intrauterine growth restriction, poor fetal growth, early onset preeclampsia (EPE), gestational hypertensive disorders, maternal hemodynamics, maternal cardiovascular function, CO, total vascular resistance (TVR), plasma volume, body water volume, maternal venous Doppler, fetal Doppler, venous hemodynamics, and volume regulation.

## Maternal Hemodynamics and Fetal Growth

Birth weight relates to many variables, such as parental anthropometrics (height, weight, BMI), race, gender, gestational age, diet, drinking habits and substance (ab)use, and medical and obstetric history (14). Poor fetal growth and, in particular, FGR may relate to a chronic state of intrauterine hypoxia resulting from preplacental, placental (e.g., abruption), and postplacental causes (e.g., cord insertion, fetal genetics; 15). Known causes of preplacental hypoxia are not only pathologic conditions, such as chronic maternal cardiovascular, pulmonary or systemic disease, anemia, and infections, but also physiological determinants, such as high altitude and maternal CO.

Cardiac output is linked mathematically to mean arterial blood pressure (MAP) and TVR according to the hemodynamics’ variant of Ohm’s law


**FIGURE 1:**
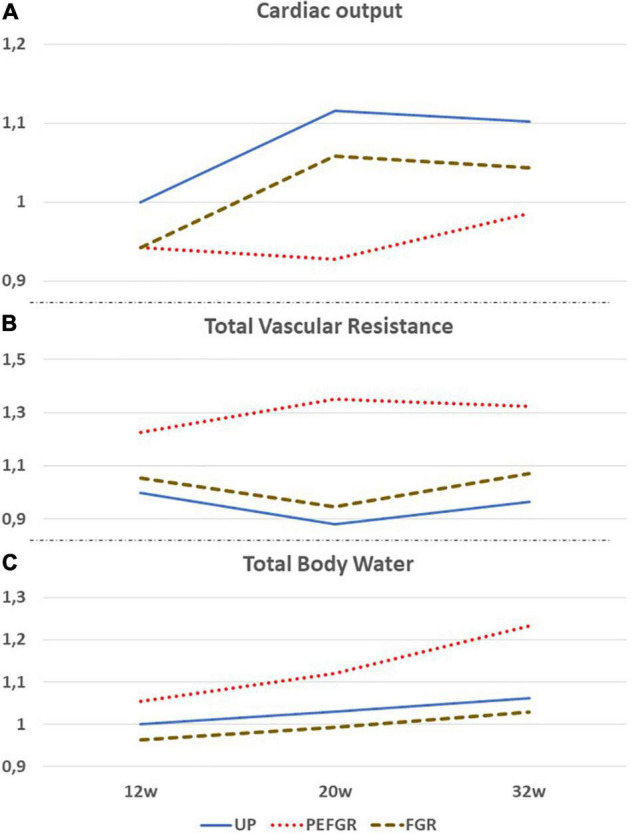
Comparative gestational evolution of maternal cardiac output **(A)**, total vascular resistance **(B)**, and total body water volume **(C)**, as measured by the bioimpedance technology between uncomplicated pregnancies (UP), normotensive fetal growth restriction (FGR), and preeclampsia with FGR (PEFGR). Values are expressed as multiples of mean first trimester values in UP. Figures adapted from 10, 11, and 28.

CO=MAP/TVR,

where CO is the product of stroke volume (SV) and heart rate (HR; 16). In the preconception period, low maternal CO, mostly in combination with increased TVR, predisposes pregnant women to gestational complications, such as PE with or without FGR (3), and the lowest CO values are observed in normotensive FGR (17). In an uncomplicated pregnancy, there is a positive correlation between CO change from preconception to mid-gestation and neonatal birth weight (18). During pregnancy from the first trimester onward, maternal CO is directly related to singleton birth weight (19–21), particularly at advanced maternal age (22), with parity (23), and multiple pregnancies (24), whereas there is an inverse relation with altitude (25) and gestational hypertensive disorders (26, 27). Figure 1 shows the gestational trends of CO and TVR as measured by the bioimpedance technology: contrary to pregnancies eventually developing PE, the evolution is similar in FGR and uncomplicated pregnancy, however, at lower CO and higher TVR (10, 11, 28). This observation has also been reported by others (29) and linked to a condition of low CO that is already present before conception (17). Low CO in FGR pregnancies mainly results from low SV (30, 31) and to a lesser degree from low HR (32, 33). Throughout an uncomplicated pregnancy, the fraction of CO deviated to the uterus doubles from 6 to 12% (34) and is achieved by an increase of (distal) internal iliac artery impedance in concert with a reduction of uterine artery impedance (35). Uterine artery blood volume flow positively correlates with birth weight (36, 37), reduces from maternal upright to the supine position with poor response to supine exercise (38), and is lower in FGR than in normal fetal growth (39). These observations all indicate that maternal cardiovascular function and uterine arterial blood supply strongly contribute to fetal growth and neonatal birth weight.

Recently, it was shown that the venous compartment and body water volume load are also involved in the regulation of fetal growth (40). Next to its metabolic functions, the liver serves as a hemodynamic organ, where a large fraction of the unstressed blood volume is stored, as a reserve volume available for an instant increase of CO by sympathetic nervous-stimulated drainage of hepatic veins in the inferior vena cava. Inverse correlations have been reported between maternal CO and birth weight, on the one hand, and hepatic venous Doppler impedance index, on the other hand (40). Next to this, in normotensive women giving birth to neonates small for gestational age (SGA), low CO was associated with low body water volume and high Doppler impedance of uterine artery and hepatic veins, all of which are indicators of a low volume circulation (10; Figure 1). These observations are in line with reported impaired expansion of maternal plasma volume in FGR pregnancies in normal (41), small, and lean (42) women. Here, it is important to mention that the reduced plasma volume, as compared to normal pregnancy, precedes a suboptimal rise of the volume regulating hormones aldosterone, progesterone, and estrogens (43). The presence of a low intravascular volume before or during pregnancy can explain why low CO and high TVR can present with normotension and why a normal gestational plasma volume expansion fails to achieve normal values of maternal CO. Contrary to normotensive FGR, EPE – which mostly is associated with poor fetal growth – low CO and high TVR present with high body water volumes already from the first trimester onwards, suggesting a different pathophysiologic background mechanism (11; Figure 1).

## Pathways of Maternal Low Volume Circulation in Early Onset Preeclampsia and Fetal Growth Restriction

In an uncomplicated pregnancy, maternal plasma volume expansion is triggered by a primary fall in systemic vascular resistance (44). The subsequent state of intravascular underfilling causes a reduction of cardiac afterload, which, in turn, is responsible for a rise in SV (45). Together with a rising HR, increased SV induces a 25% increase in CO at 6 weeks of gestation (17). Due to anatomic and physiologic properties of the high volume/low resistance of pulmonary circulation, increased CO results in a reduction of pulmonary vascular resistance *via* capillary recruitment and distention (46). In pregnant women, increased CO and the associated enlarged pulmonary capillary bed are responsible for a rise of intrathoracic water as measured by bioimpedance technology as early as 6–7 weeks of gestation (47, 48).

**FIGURE 2:**
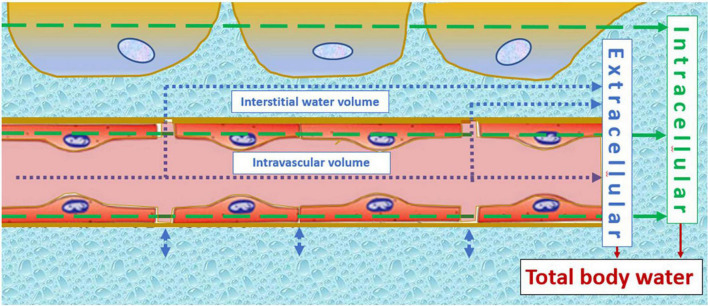
Illustration of the constant fluid exchange between intravascular and interstitial volumes at the level of the microcirculation. This interconnectivity indicates that plasma volume changes cannot be interpreted without knowledge of interstitial water volume, or indirectly *via* measurement of total body water volume. Normotensive fetal growth restriction relates to a constitutionally low intravascular volume, remaining lower in uncomplicated pregnancies despite normal gestational volume expansion. Early onset preeclampsia, on the other hand, is a state of increased adrenergic activation (155), shifting blood from the venous capacitance bed into the circulation and despite this, cardiac output fails to rise. This can only be explained by a shift from the intravascular compartment to the interstitium, which is in line with clinical signs, such as malleolar or pulmonary edema, in early onset preeclampsia. The arrows indicate the principle of volume estimation by bioimpedance spectrum analysis of intracellular (green) and extracellular (blue) compartments, the latter being the sum of intravascular and interstitial volumes.

Meanwhile, SV and left atrial dimensions continue to rise in normal pregnancies; this is not true for pregnancies destined to develop severely impaired fetal growth (45) and/or EPE (33). As shown in Figure 1, CO is persistently lower than normal in normotensive FGR despite a normal gestational rise. In EPE, however, CO fails to rise after 8 weeks of gestation (17), and this is associated with the echocardiographic reduced left atrial area and fractional area change (33), as well as increased left ventricular end-systolic and end-diastolic volumes (49). This pathophysiologic condition is responsible for the increase of left atrial filling pressure, with the subsequent retrograde rise of pulmonary venous pressure and capillary hydrostatic pressure, resulting in exudation of intravascular fluids similar to the mechanisms observed in heart failure (50). A rise of pulmonary interstitial fluids before the clinical presentation of symptomatic pulmonary edema is a known phenomenon in chronic heart failure, and the detection of this condition by bioimpedance technology is useful to predict and timely counteract the severity of pulmonary edema (50, 51). As shown in Figure 1, the same mechanism is likely to occur in early preeclampsia (EPE): an asymptomatic exudation of intravascular fluids in the pulmonary interstitium can precede the development of edema elsewhere in the female body and explains the combination of increased total body water volume without the concomitant rise of CO in EPE. The constant exchange between intravascular and interstitial volumes is a normal physiologic function of the microcirculation and indicates that abnormal changes in plasma volume cannot be interpreted correctly without considering changes in other body volume compartments. This phenomenon is illustrated visually in Figure 2. The nature of this pathophysiologic pathway is in line with the reported increased serum concentrations of atrial natriuretic peptide (52) and copeptin/vasopressin in PE (53) and with the impaired expansion of maternal plasma volume in EPE (9). The early gestational onset of this phenomenon is also supported by the shallow, but significant, rise of serum hemoglobin concentrations and hematocrit in the first trimester of pregnancies destined to develop EPE (31, 54, 55) but not in those eventually leading to FGR (56). As explained above and illustrated in Figure 1, FGR pregnancies show a normal rise of plasma volume and total body water, superimposed upon constitutionally low body water already present before conception.

## Laboratory and Clinical Experiments Supporting Maternal Low Volume Circulation in Fetal Growth Restriction

A low sodium diet for pregnant rats prevents normal maternal plasma volume expansion and induces FGR and low placental weight (57). This association presents with poor dilatation of the uterine and radial arteries, together with reduced uterine blood volume flow and increased vascular resistance, and the activation of the renin-angiotensin-aldosterone system with reduced expression of placental Angiotensin II receptor subtype 1 (58) and with increased concentrations of placental markers of hypoxia (57, 58). Murine FGR presents with a reduced placental blood flow rate, demonstrable by both contrast-enhanced sonography and magnetic resonance imaging (MRI; 59).

Different types of MRI technology have been proven useful for non-invasive studies of placental perfusion in animals and human (60), allowing for the quantification of blood flow volume (61), and mapping and fractional differentiation of perfused and non-perfused areas (62, 63). As compared to normal pregnancies, FGR presents with a one-third decrease of placental perfusion fraction (64), strongly correlating with increased Doppler impedance measurements of uterine and umbilical arteries, particularly of the ductus venosus (DV; 64, 65). Blood flow velocity in the FGR placenta is reduced, is non-homogenous, and shows intermittent stops in severe cases with increased DV pulsatility index (65). Taken together, all MRI placenta perfusion studies show evidence for underfilling of the intervillous space, which is a trigger for both maternal and fetal reflex responses. Laboratory and animal models show that incomplete spiral artery trophoblast invasion results in an increase of oxygen tension (66) and perfusion pressure (67) in the intervillous space, with constriction of maternal chorionic plate venules (68), enhanced myogenic activity of uterine and radial arteries (69) despite an activated pathway of nitric oxide (NO)-dependent vasodilation (70), and altered placental cell populations and trophoblast differentiation (71).

Poor placenta perfusion in both PE and FGR is supported by histologic signs, such as accelerated villus branching, large and numerous syncytial knots, and small sclerotic villi, suggestive of placental hypoxia and/or oxidative stress (72).

## Epidemiologic Data Supporting Maternal Low Volume Circulation in Fetal Growth Restriction

Epidemiologic studies have shown an intergenerational association of FGR: women who, themselves, were born with low birth weight are more likely to reproduce low birth weight offspring (73–75). Apart from genetic, familial, and socioeconomic predispositions, complex molecular processes, such as genetic imprinting, microchimerism, and epigenetic modifications, are involved (76, 77), and this results from early neonatal life onward in the disruption of endocrine and metabolic systems (78, 79) together with permanent dysfunctions of vital organ systems (80, 81), such as the kidneys (82, 83), the heart and blood circulation (76, 84, 85), the endothelium (86), and the immune system (80). In the long run, these systemic dysfunctions predispose to early onset adult disease (87). Body water volume homeostasis is another system that is dysfunctional in FGR, involving the renin-angiotensin system (88) and natriuretic peptides (89, 90). A particularly interesting observation is that FGR predisposes to low plasma volume in adult life (91). In former preeclamptic women, it was shown that preconceptional low plasma volume predisposes to recurrence of PE (92). Even more important is that, in nulliparous women, preconceptional vascular dysfunction (93) and angiotensinogen phenotype-dependent low plasma volume (94) predispose to abnormal perinatal outcomes (95). Low plasma volume coexists with poor venous reserves, resulting from abnormal venous capacitance and vascular compliance together with autonomic nervous dysfunction (96), and is associated with recurrent first-trimester pregnancy loss (97). As explained in the section, combined venous hemodynamic dysfunction in both mother and fetus in PE with FGR has also been observed in studies using Doppler-ECG ultrasonography.

## Interactive Maternal-Fetal Hemodynamics in Fetal Growth Restriction

### Mother-to-Fetus Circulatory Interactions in Fetal Growth Restriction

Placental angiogenesis and vasculogenesis involve cell-communicating factors including vascular endothelial growth factor (VEGF), placental growth factor (PlGF), and oxygen (98). Placental histology studies have shown that FGR is characterized by decreased branching angiogenesis with increased apoptosis, resulting in a reduced number of terminal villi and stromal capillaries, poor villi vascular density, fewer intervillous pores, increase of intervillous thrombi, villous infarctions, villitis, and thickening of the basal membrane (99, 100), finally resulting in overall reduced exchange surface area (101–103). This is associated with a lower and higher release of VEGF-A and PlGF, respectively, an unbalanced production of their cell receptors, and the release of anti-angiogenic factors, mechanisms supported by reduced oxygen tension and volume flow (39, 98). As a result, endothelial cells from the FGR placenta show dysregulated biochemical signaling with failed compensatory response to resist high blood flow (104), demonstrable by high Doppler impedance measurements at the level of uterine and umbilical arteries (15). Doppler sonography is a useful method to assess uterine and umbilical artery flow impedance. Increased uterine artery Doppler pulsatility index and resistivity index have long been considered a result of abnormal spiral artery adaptation at implantation, but, currently, increasing number of experimental, clinical, and epidemiological data are in favor of the opposite pathway, suggesting abnormal placentation merely as the result rather than the cause of abnormal uterine perfusion (105, 106). In this context, the inverse correlation between preconceptional uterine artery Doppler impedance measurements and subsequent birthweight in formerly preeclamptic women is illustrative (107).

With advancing gestation of FGR, the intrauterine environment becomes more and more hypoxic, to which the fetus responds by redirecting the blood supply, preferably to vital organs like the heart and the brain, at the expense of subdiaphragmatic organ perfusion (15). Fetal brain sparing can be documented by Doppler flow measurements at the level of umbilical and cerebral arteries with the calculation of relative impedance ratios (108–110). Associations have been reported between abnormal uterine-fetal Doppler measurements and maternal hemodynamic dysfunction (8) and between fetal cerebral Doppler changes and adverse outcomes (111).

**FIGURE 3:**
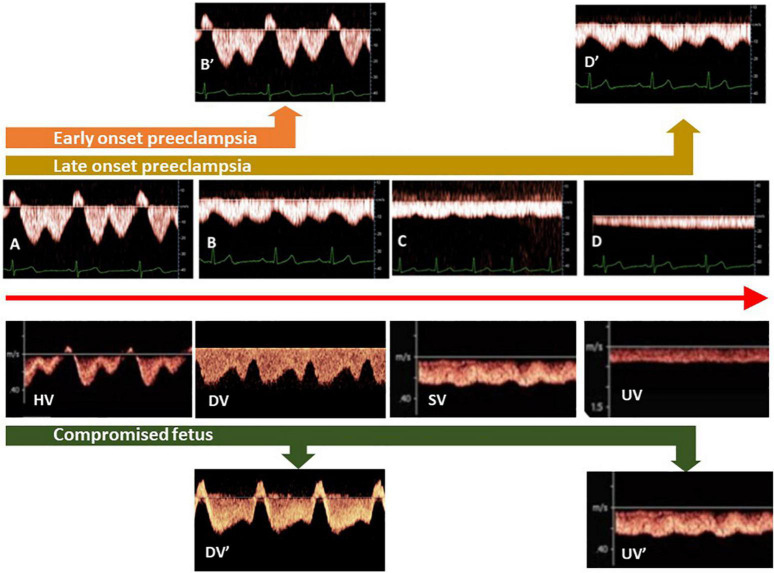
Different types of venous Doppler waveforms in maternal and fetal circulations, varying between triphasic **(A)**, biphasic **(B)**, and monophasic **(C)** to flat **(D)**. In the fetus and non-pregnant adults, triphasic patterns are found close to the heart [hepatic veins (HV)], whereas flat patterns are present at distant locations [limbs, umbilical veins (UV)]. During uncomplicated pregnancy, HV patterns shift from **(A–D)**. In the clinical stage of preeclampsia with FGR, biphasic HV patterns become triphasic (orange arrow), whereas in term preeclampsia, monophasic patterns evolve to biphasic (brown arrow). In ductus venosus (DV) of FGR fetuses, a shift from biphasic to triphasic patterns occurs simultaneously with deteriorating fetal condition (green arrow). (SV: fetal splenic vein).

On top of fetal arterial Doppler flow changes, DV Doppler flow patterns, shifting from biphasic to triphasic, offer additional information on deteriorating fetal condition (112–114; Figure 3). This evolution is very similar to the change of Doppler flow patterns in the maternal hepatic vein from early pregnancy to the clinical stage of EPE (115; Figure 3). The sequence of venous Doppler waveform changes, however, is different between the FGR fetus and the woman with EPE. In FGR, DV Doppler flow is secondary to altered cardiac function due to increased afterload for the right ventricle (RV) but not the left one, with subsequently reduced RV compliance and increased right atrium filling pressure, which reflects in the reversed DV Doppler A-wave (116, 117) and reduced CO to the placenta (118). In EPE, triphasic HV Doppler flow patterns are already present weeks before the clinical onset of disease (119), indicating the involvement of venous vascular wall activity (120, 121). Further research should elucidate whether this difference relates to a different intravascular filling state, which is low for the woman with PEFGR (Figure 1) and is linearly related to birth weight, irrespective of preceding intrauterine fetal condition (122, 123).

### Conceptus-to-Mother Circulatory Interactions in Fetal Growth Restriction

An important fetal-maternal communication system is the intravascular shedding of placental particles, varying in size and shape between multinucleated syncytial aggregates and subcellular nanovesicles, originating from not only apoptosis in a normal pregnancy but also necrosis in PE (124), and is associated with increased serum total cell-free DNA (125). These particles act *via* intravesical molecules and micro-RNA or circular RNA that, after phagocytosis by endothelium and immune cells, is capable of inducing sterile inflammation *via* increased surface expression of monocyte adhesion receptors, such as E-selectin, secretion of pro-inflammatory cytokines, namely interleukin 6 and transforming growth factor β (124). Downregulation of specific micro-RNAs has been reported in FGR, some of which are shared with PE (126). Next to this, the production of mediators of angiogenesis and vasoactivity is stimulated in FGR, such as soluble FMS-like tyrosine kinase and VEGF (127), whereas pregnancy-associated placental protein A (PAPP-A) is reduced (128). Many other vasoactive and immunologic mediators have also been studied in maternal serum concerning diagnosis or prediction of PE and/or FGR. The most-reported biomarkers studied for this purpose are summarized in Table 1. It has generally been accepted that the placenta is the primary source of these factors and, as such, is the primary driver of the global functioning of the maternal circulation. It should be emphasized, however, that abnormal serum concentrations of many of these factors have also been documented in non-pregnant individuals with (pre-clinical) chronic cardiovascular and/or renal disease (Table 1). This indicates that, apart from some placenta-specific products, it cannot be concluded indisputably whether the origin of the vasoactive and/or immunomodulatory serum substances associated with PE ± FGR is placental, maternal, or combined.

**TABLE 1:** Serum markers of fetal growth restriction (FGR), preeclampsia (PET), and/or cardiovascular disease (CVD).

	Physiologic function	Pregnancy		Non-pregnant		References		
		FGR	PE		Type CVD	FGR	PE	CVD
CRP	Immunomodulation	↑	↑	↑	CHD, HF	(156)	(157)	(158)
VEGF	Pro-angiogenic Pro-vasculogenic	↓	↓	Polymorfisms	CHD	(98)	(159)	(160)
sFLT-1	Anti-angiogenic	↑	↑	↑	CHD, HF	(127)	(127)	(161)
sEng	Anti-angiogenic	↑	↑	↑	CHD	(162)	(159)	(163)
Activin A	Immunomodulation Apoptosis	↑	↑	↑	CHD, HT	(164)	(165)	(166)
Leptin	Immunomodulation Angiogenic	↑	↑	↑	CHD	(167)	(168)	(169)
sE-selectin	Immunomodulation	↑	↑	↑	HT	(170)	(171)	(172)
ADAM 12	Angiogenic Immunomodulation	↓	↓	↓	HF	(173)	(108)	(174)
ADMA	Vasodilatation	↑	↑	↑	CHD, HF, HT	(175)	(176)	(177)
PLGF	Pro-angiogenic	↓	↓	↑	CHD, HF	(127)	(159)	(161)
PAPP-A	Lysis IGF-BP	↓	↓↑↑	↑	CHD	(128)	(178)	(179)
ADM	Pro-angiogenic	↓	↓	↑	AMI	(179)	(180)	(181)

*Abbreviations: FGR, fetal growth restriction; PE, preeclampsia; CVD, cardiovascular disease; ↑, high serum concentration; ↓, low serum concentration; ↓↑↑, cerum concentration changing from low to high; CHD, coronary heart disease; HT, hypertension; HF, heart failure; AMI, acute myocardial infarction; CRP, C-reactive protein; VEGF, vascular endothelial growth factor; sFlt-1, soluble fms-like tyrosine kinase 1; sEng, soluble endoglin; sE-selectin, soluble E-selectin; ADAM 12, A disintegrin and metalloproteinase 12; ADMA, asymmetric dimethylarginine; PLGF, placental Growth Factor; PAPP-A, pregnancy associated placental protein A; and ADM, adrenomedullin.*

## Clinical Implications of Maternal Low Volume Circulation in Fetal Growth Restriction

The association between low maternal volume circulation and FGR has important clinical implications. First, maternal hemodynamics can offer additional information in unexplained cases of FGR, where all other known etiologic factors have been excluded (129). In recent times, non-invasive assessment of CO and peripheral resistance is feasible by different types of technologies (130), which, when used with appropriate reference ranges under standardized conditions (8, 131), can easily identify those women with a low output/high resistance circulation who are particularly at risk for FGR. This information is not only useful for the diagnosis of FGR but can also be of value from the first trimester onward before poor fetal growth is evident (29, 45, 132). This opens perspectives toward the implementation of maternal hemodynamics parameters into current screening programs for FGR (7, 133). Preliminary, though promising, data on the reduction of FGR have been reported on the supplementation of the screen positive high-risk group with antiplatelet therapy (134, 135) and/or the antioxidants lycopene or L-Arginin (136).

Secondly, maternal low volume circulation can be a target for the prevention of FGR pregnancies. Physical exercise is a well-known useful intervention for the improvement of cardiovascular functions (137). In formerly preeclamptic women included in a program of controlled physical exercise, an increase in stroke and plasma volume was observed (138) together with the improvement of venous reserves up to the pretraining levels of controls (139). In overweight and obese pregnant women, physical exercise throughout gestation was associated with a lower incidence of gestational diabetes and reduced third-trimester systolic blood pressure (140). Lower systolic blood pressure was also observed in trained versus non-trained normotensive pregnant women (141). Importantly, maternal physical training was shown to influence fetal cardiovascular functions by the increase of left ventricular output and aortic peak flow velocity (142) and by lower carotid artery wall thickness in offspring (143).

A third important implication for pregnancies complicated with both FGR and hypertension is the antihypertensive therapy of choice in addition to low dose aspirin (135) initiated before 16 weeks at ≥100 mg PD (134). Blood pressure-lowering pharmacologic mechanisms are different between beta-blockers, calcium blockers, and centrally active agents due to which the effects on neonatal birth weight are different. There is a growing body of evidence that adrenergic beta-blockers are associated with an increased birth rate of neonates SGA (144–148) with a mean effect estimated at ≤200 *g* at term (149). A similar but less pronounced effect has also been reported for Alfa-MethylDopa (145, 150) but not for calcium channel blockers (145). A possible explanation for this differential effect is that beta-blockers partially exert their effects *via* a reduction of CO (151), whereas calcium channel blockers mainly function *via* reduced peripheral resistance with a compensatory rise of CO (152). As such, from a theoretical perspective, calcium channel blockers might be a better choice than beta-blockers with respect to avoiding a negative pharmacologic impact on fetal growth. There, however, is an urgent need for more fundamental and clinical research into the differential mechanisms and outcomes of antihypertensive therapies in pregnant women.

An interesting clinical confirmation of the association between maternal intravascular volume and the gestational outcome has been reported in two studies by the Tor Vergata university of Rome’s research team (153, 154) using the potently vasodilating NO donors. As compared to a historical control group, a cohort of 26 FGR pregnancies treated with NO donors and plasma volume expansion showed an improvement in maternal CO and TVR and in higher birth weight after 2 weeks (153). Similarly, in 32 women with hypertension and FGR with absent end-diastolic umbilical Doppler flow, randomized between conventional management with or without NO donors and plasma volume expansion, the reappearance of diastolic umbilical blood flow and the prolongation of gestation were observed in the treated group (154).

## Perspectives

This review summarizes evidence from clinical, experimental, and laboratory observations on the association between low volume maternal circulation and poor fetal growth. Conditions of low maternal CO can present before conception or develop during the earliest stages of pregnancy, in conditions of both normotension or hypertension relative to the balance between flow volume and vascular resistance. Intravascular volume is intimately related to CO, renal function, and through aldosterone and the renin-angiotensin system to peripheral vascular resistance and blood pressure. Hence, acknowledging that the association between maternal intravascular filling state and fetal wellbeing opens perspectives toward prevention, management, and reduction of intergenerational transfer of poor fetal growth. However, more in-depth exploration is needed on the role of normal or abnormal maternal cardiovascular function in obstetric and neonatal outcomes.

## Author Contributions

Both authors listed have made a substantial, direct, and intellectual contribution to the work, and approved it for publication.

## Conflict of Interest

The authors declare that the research was conducted in the absence of any commercial or financial relationships that could be construed as a potential conflict of interest.

## Publisher’s Note

All claims expressed in this article are solely those of the authors and do not necessarily represent those of their affiliated organizations, or those of the publisher, the editors and the reviewers. Any product that may be evaluated in this article, or claim that may be made by its manufacturer, is not guaranteed or endorsed by the publisher.

## Abbreviations

CO: cardiac output
DV: ductus venosus
EPE: early onset preeclampsia
FGR: fetal growth restriction
HR: heart rate
HV: hepatic veins
IL6: interleukin 6
MAP: mean arterial pressure
PAPP-A: pregnancy associated placental protein A
PE: preeclampsia
PlGF: placental growth factor
RV: right ventricle
S-FLT 1: soluble FMS-like tyrosine kinase 1
SGA: small for gestational age
SV: stroke volume
SV (Figure 3): splenic vein
TGF β: transforming growth factor β
TVR: total vascular resistance
UV: umbilical vein
VEGF: vascular endothelial growth factor.
